# Dyskeratosis congenita with a novel genetic variant in the *DKC1* gene: a case report

**DOI:** 10.1186/s12881-018-0584-y

**Published:** 2018-05-25

**Authors:** Vithiya Ratnasamy, Suganthan Navaneethakrishnan, Nirmala Dushyanthi Sirisena, Nana-Maria Grüning, Oliver Brandau, Kumanan Thirunavukarasu, Casey L. Dagnall, Lisa J. McReynolds, Sharon A. Savage, Vajira H. W. Dissanayake

**Affiliations:** 1 University Medical Unit, Teaching Hospital Jaffna, Jaffna, Sri Lanka; 2 Human Genetics Unit, Faculty of Medicine, University of Colombo, Kynsey Road, Colombo 8, Sri Lanka; 3 Centogene AG, Schillingallee 68, 18057 Rostock, Germany; 4 Cancer Genomics Research Laboratory, Leidos Biomedical Research, Inc., Frederick National Laboratory for Cancer Research, Frederick, Maryland USA; 5 Clinical Genetics Branch, Division of Cancer Epidemiology and Genetics, National Cancer Institute (NCI), Bethesda, Maryland USA

**Keywords:** *DKC1*, Dyskeratosis congenita, Nail dystrophy, Oral leukoplakia, Pancytopenia, Skin pigmentation

## Abstract

**Background:**

Dyskeratosis congenita (DC) is a rare genetic disorder of bone marrow failure inherited in an X-linked, autosomal dominant or autosomal recessive pattern. It has a wide array of clinical features and patients may be cared for by many medical sub specialties. The typical clinical features consist of lacy reticular skin pigmentation, nail dystrophy and oral leukoplakia. As the disease advances, patients may develop progressive bone marrow failure, pulmonary fibrosis, oesophageal stenosis, urethral stenosis, liver cirrhosis as well as haematological and solid malignancies. Several genes have been implicated in the pathogenesis of dyskeratosis congenita, with the dyskerin pseudouridine synthase 1 (*DKC1*) gene mutations being the X-linked recessive gene.

**Case presentation:**

Herein, we report a 31-year-old male with history of recurrent febrile episodes who was found to have reticulate skin pigmentation interspersed with hypopigmented macules involving the face, neck and extremities, hyperkeratosis of palms and soles, nail dystrophy, leukoplakia of the tongue, premature graying of hair, watery eyes and dental caries. Several of his male relatives, including two maternal uncles and three maternal cousins were affected with a similar type of disease condition. Pedigree analysis suggested a possible X-linked pattern of inheritance. Genetic testing in the proband showed a novel hemizygous, non-synonymous likely pathogenic variant [NM_001363.4: c.1054A > G: p.Thr352Ala] in the PUA domain of the *DKC1* gene. Quantitative polymerase chain reaction for relative telomere length measurements performed in the proband showed that he had very short telomeres [0.38, compared to a control median of 0.71 (range 0.44–1.19)], which is consistent with the DC diagnosis. Co-segregation analysis of the novel mutation and telomere length measurements in the extended family members could not be performed as they were unwilling to provide consent for testing.

**Conclusions:**

The novel variant detected in the *DKC1* gene adds further to the existing scientific literature on the genotype-phenotype correlation of DC, and has important implications for the clinical and molecular characterization of the disease.

## Background

Dyskeratosis congenita (DC), also known as Zinsser Cole-Engman syndrome, is a rare genetic disorder with multi-system involvement and reduced survival. At least 12 genes have been implicated in the pathogenesis of DC, with the dyskerin pseudouridine synthase 1 (*DKC1*) gene mutations being the commonest. DC is inherited in an X-linked, autosomal dominant or autosomal recessive pattern. Majority of DC cases are X-linked and caused by pathogenic variants in the *DKC1* gene (20–25%). However, in 20–30% of cases no variants can be identified [1]. *DKC1* encodes the peptide dyskerrin, which is responsible for ribosome biogenesis and telomere maintenance. Germline mutations in telomere biology genes result in aberrantly short telomeres and progressive cell death and chromosomal instability [1]. DC is clinically characterized by the clinical triad of lacy reticular skin pigmentation mainly involving the neck and upper anterior chest, nail dystrophy and oral leukoplakia. The clinical spectrum includes various presentations with bone marrow failure, pulmonary fibrosis, liver failure, oesophageal stenosis, urethral stenosis, as well as eye abnormalities (epiphora, blepharitis, sparse eyelashes, ectropion, entropion, trichiasis), dental caries, periodontal disease and taurodontism [2]. Patients with DC are also at increased risk of developing hematological and solid tumours including myelodysplastic syndrome, acute myelogenous leukaemia, squamous cell carcinoma of head/neck and anogenital cancer [3]. The premature mortality in DC is mainly due to bone marrow failure, and the only curative treatment is haematopoietic stem cell transplantation [4]. In this report, we describe a case of DC with X-linked pattern of inheritance who was found to have a novel hemizygous, non-synonymous likely pathogenic variant in the *DKC1* gene.

## Case presentation

**Fig. 1:**
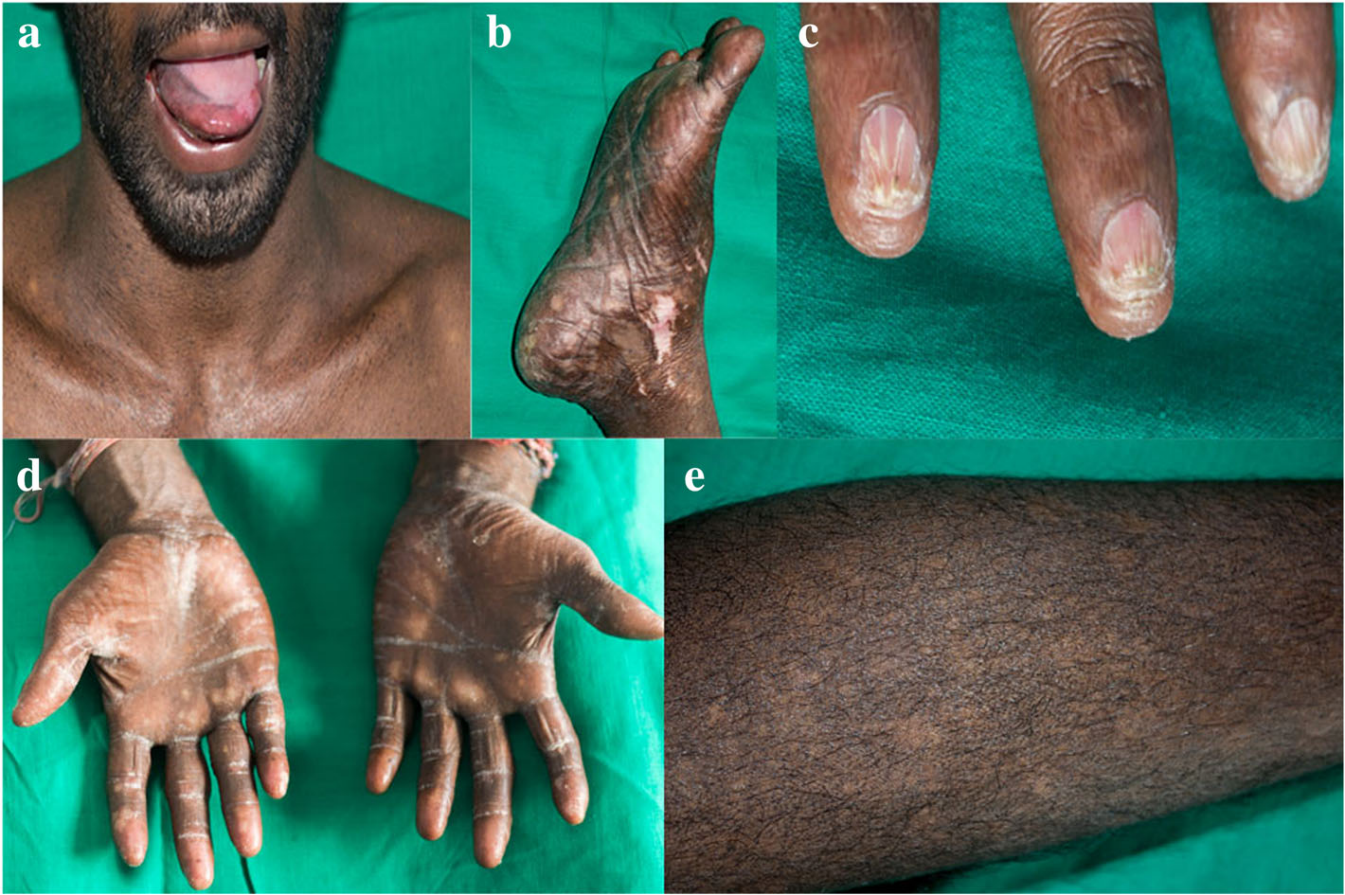
Clinical features of the proband with dyskeratosis congenita showing: **a** oral leukoplakia, reticular hyperpigmentation of the neck and upper chest, **b** hypopigmented patches on the leg, **c** dysplastic finger nails, **d** adermatoglyphia, **e** lacy reticular hyperpigmentation of the lower limb

A 31-year-old male from Udupiddy Jaffna, in the Northern Province of Sri Lanka, presented to the medical casualty with fever of 1 week duration as his first ever medical consultation. He gave a history of recurrent febrile episodes which resolved spontaneously over the preceding 2 months. Clinical examination showed the presence of reticulate skin pigmentation interspersed with hypopigmented macules involving the face, neck and extremities, hyperkeratosis of palms and soles and nail dystrophy (Fig. 1), which he claimed to have had since the age of 15-years. He had noticed progressive worsening of the skin changes since then. In addition, he had leukoplakia of the tongue, premature graying of hair, watery eyes and dental caries.

**Fig. 2:**
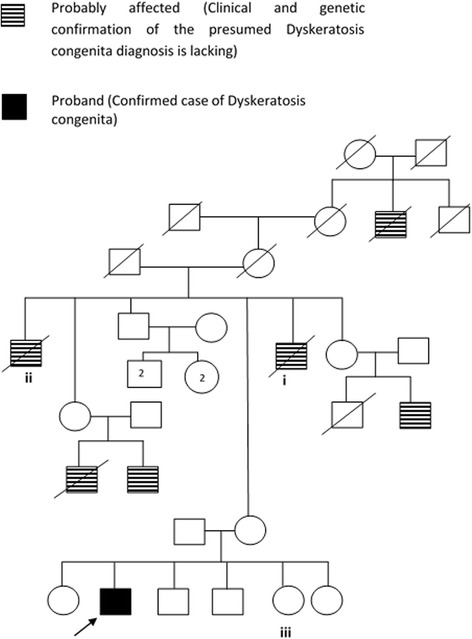
Pedigree constructed from the details provided by the proband’s mother: i) proband’s maternal uncle died due to a febrile illness at the age of 26 years, ii) proband’s maternal uncle died at the age of 50 years due to tuberculosis, iii) proband’s sister is a probable carrier with history of similar localised skin pigmentation and nail changes but no history of significant illnesses or recurrent infections

He was a product of a non-consanguineous marriage. Several of his maternal male relatives, including two deceased maternal uncles and three maternal cousins were reported to be affected with a similar disease condition (Fig. 2). One of his sisters had similar localized skin pigmentation in the hands and nail changes. However, clinical and genetic confirmation were lacking in the family members. Analysis of the pedigree constructed from the proband’s mother’s recollections suggested a possible X-linked pattern of inheritance. Clinical diagnosis of DC was made based on the classical clinical features at presentation. Initial investigations showed pancytopenia (Total white blood cells: 2.48 × 10^3^/μl [4 – 11 × 10^3^/μl], neutrophils: 1.39 × 10^3^/μl [2 - 7 × 10^3^/μl], lymphocytes: 0.44 × 10^3^/μl [0.8 - 4 × 10^3^/μl], monocytes: 0.61 × 10^3^/μl [0.12 -1.2 × 10^3^/μl], red blood cells: 3.24 × 10^6^/μl [3.5 - 5.5 × 10^6^/μl], platelets: 90 × 10^3^/μl [150 - 450 × 10^3^/μl]), ESR: 60 mm/1st hour [0 - 22 mm/1st hour] and C - reactive protein: 83 mg/l [0 - 6 mg/l]. He had a macrocytic anemia with a haemoglobin level of 11.3 g/dl [13.5 - 17.5 g/l] and Mean Corpuscular Volume of 101.6 fl [80 - 96 fl]. Blood film showed pancytopenia. Bone marrow aspiration and biopsy confirmed bone marrow failure. Ultrasound scan of the abdomen showed splenomegaly of 13 cm. Initial chest radiograph was unremarkable.

Written informed consent was obtained from the patient for the genetic analysis. *DKC1* gene was analyzed by polymerase chain reaction (PCR) amplification and sequencing of both DNA strands of the entire coding region and conserved exon-intron splice junctions. A previously unreported hemizygous, non-synonymous likely pathogenic variant (NM_001363.4: c.1054A > G: p.Thr352Ala) was identified. This variant was predicted as probably damaging using the software analysis tools Provean, Mutation Taster and Align-GVGD. It is located in a moderately conserved nucleotide and highly conserved amino acid position with small physiochemical differences between the exchanged amino acids (Alamut v.2.7.1). It resides in a functionally important domain of the *DKC1* gene known as the PUA domain, near Ala353Val which is a common hot spot of the *DKC1* mutations. This novel variant was not found in the Exome Aggregation Consortium database (which comprises approximately 60,000 subjects), or in any of the other population specific databases (1000 Genomes). It was also absent in our existing 50 de-identified Sri Lankan exome/genome sequences.

**Fig. 3:**
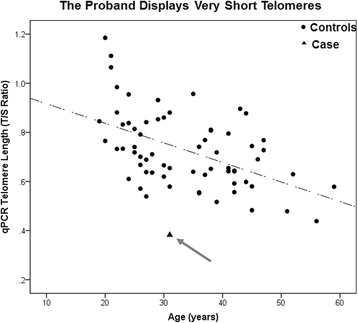
Proband has short telomeres relative to age matched controls. qPCR telomere length assay was performed using genomic DNA as a template on the proband and 66 controls of variable ages. Telomere length is plotted versus age in years. ● = controls ▲ = proband, marked with an arrow

Genetic counseling and familial co-segregation analysis in the mother, unaffected brothers and sisters, maternal sisters and their affected sons, and affected maternal uncles could not be done as the family members were unwilling to provide consent for genetic testing. DC is also commonly confirmed by molecular diagnosis when a patient’s peripheral blood mononuclear cell telomere length is shorter than 99% of the age-matched population (i.e. below the 1 percentile) or by determining decreased telomerase RNA levels. Quantitative PCR (qPCR) for relative telomere length measurements was performed in the proband (extended family members were unwilling to undergo testing). Relative telomere length determination by qPCR measures the ratio of telomere (T) signals, specific to the telomere hexamer repeat sequence TTAGGG, to autosomal single copy gene (S) signals. This ratio is normalized by control DNA samples to yield relative standardized T/S ratios proportional to average telomere length. In this technique reactions are performed independently, so a standard curve of pooled genomic DNA samples is utilized to assess the amount of each signal, while compensating for inter-plate variations in PCR efficiency. This telomere length measurement assay was adapted from the published method by Cawthon [4]. The results showed that his relative telomere length (T/S ratio) was 0.38, compared to a control median of 0.71 (range 0.44-1.19) by qPCR, indicating that the patient, indeed, had very short telomeres, which is consistent with the DC diagnosis (Fig. 3).

Functional characterization of the novel *DKC1* variant to determine the effects of the variant on protein level could not be performed as the resources needed to conduct such complex and highly technical assays were not readily available at our centre. However, based on the data outlined above and the typical phenotypic features in the proband along with the strong family history suggestive of X-linked inheritance pattern, it is likely that hemizygosity for the novel variant resulted in DC in this patient. Repeat bone marrow biopsy done 3 months later confirmed the diagnosis of evolving bone marrow failure, compatible with a congenital bone marrow failure syndrome. He was re-admitted 3 months after the initial diagnosis with 1 week history of fever, and clinical features of pneumonia. His disease took a rapidly progressive course with severe dysphagia and vomiting developing over 3 weeks. In addition, he developed severe pancytopenia with acute liver failure and succumbed to his illness 4 months after the initial diagnosis.

## Discussion and conclusions

In the diagnostic evaluation of this patient who presented with fever and pancytopenia, the initial differential diagnoses were DC or Fanconi anemia. However, with the classical clinical triad of lacy reticular skin pigmentation, oral leukoplakia and nail dystrophy, absence of bone abnormalities, and a strong family history, he was diagnosed as having DC. Hoyeraal Hreidarsson syndrome and Revesz syndrome are two severe forms of DC which manifest in early childhood. Hoyeraal Hreidarsson syndrome is characterized by the presence of clinical features of DC along with cerebellar hypoplasia, immunodeficiency, intra uterine growth retardation and developmental delay [5]. Revesz syndrome is diagnosed in the presence of bilateral exudative retinopathy with typical features of DC [6]. None of the features of the above two syndromes were found in our patient.

Germline mutations in key components of telomere biology result in the extremely short telomeres characteristic of DC. Several genes have been identified as being responsible for this syndrome including: *DKC1,* CTS Telomere Maintenance Complex Component 1 (*CTC1)*, Regulator of Telomere Elongation Helicase 1 (*RTEL1*), TERF 1 Interacting Nuclear Factor 2 (*TINF2*), Telomerase RNA Component (*TERC*), Telomerase Reverse Transcriptase (*TERT*) [1], Adrenocortical Dysplasia Homolog (*ACD*) [7], NHP2 Ribonucleoprotein (*NHP2*) [8], NOP 10 Ribonucleoprotein (*NOP10*) [9], Poly(A)-specific Ribonuclease (*PARN*) [10], Nuclear assembly factor 1 *(NAF1)* [11] and WD Repeat Containing Antisense to TP53 (*WRAP53* (*TCAB1*)) [12]. Among these, *DKC1* mutations are the most common [1]. Nearly 40 pathogenic variants have so far been reported in the *DKC1* gene [1]. Our patient, with classical clinical features, very short telomeres, and a history of similar disease condition among his relatives, fulfilled the criteria for DC [13]. X-linked pattern of inheritance was observed from his pedigree analysis, however clinical and genetic confirmation of the presumed DC diagnosis was lacking among his extended family members. It has been reported that patients with X-linked pattern of inheritance commonly have mutations in the *DKC1* gene [14], encoding the peptide dyskerrin, which is responsible for ribosome biogenesis and telomere maintenance [15]. It has also been reported that carrier females with pathogenic *DKC1* variants may be mosaics and may manifest cutaneous and parenchymal telomere phenotypes due to X-chromosome skewing. Even though one of his sisters had similar localized skin pigmentation in the hands, clinical or genetic confirmation of her carrier status could not be ascertained to corroborate the preceding statement regarding mosaic female carriers, as she was unwilling to provide consent for genetic testing [16].

This patient presented for his first ever medical consultation at a relatively advanced stage, despite the strong family history of an undiagnosed hereditary disease and early deaths among male relatives. Even though he was worried about his cosmetic appearance, he was reluctant to seek medical attention due to lack of awareness and the fear of social stigma. Even mild depression may adversely affect a patient’s willingness to seek medical attention and enthusiasm for regular follow up [17], especially in the Sri Lankan context. This is an additional challenge and concern in the treatment of DC, highlighting the need for psychological support and the important role of genetic counseling in helping the family members to clarify their genetic risk status and make informed decisions. Primary care physicians, dentists, and dermatologists should be vigilant enough to diagnose this rare condition in their clinical practice, where patients may present with recurrent febrile illnesses, dental problems, and skin or nail changes for routine evaluation. Psychological and socio-cultural issues are often overlooked and should be addressed in the overall management of DC. The novel variant identified here in the *DKC1* gene adds further to the existing scientific literature on the genotype-phenotype correlation of DC and has important implications for the clinical and molecular characterization of the disease condition.

## Abbreviations

DC: Dyskeratosis congenita
*DKC1*: Dyskerin pseudouridine synthase 1
PCR: Polymerase chain reaction
qPCR: Quantitative polymerase chain reaction
